# *Staphylococcus aureus* Bacteriophage Suppresses LPS-Induced Inflammation in MAC-T Bovine Mammary Epithelial Cells

**DOI:** 10.3389/fmicb.2018.01614

**Published:** 2018-07-23

**Authors:** Lili Zhang, Xiang Hou, Lichang Sun, Tao He, Ruicheng Wei, Maoda Pang, Ran Wang

**Affiliations:** Key Laboratory of Food Quality and Safety of Jiangsu Province-State Key Laboratory Breeding Base, Institute of Food Safety and Nutrition, Jiangsu Academy of Agricultural Sciences, Nanjing, China

**Keywords:** *Staphylococcus aureus*, bacteriophage, inflammation, LPS, NF-κB, MAC-T cells

## Abstract

Several previous studies have shown that bacteriophages can significantly affect the production of various cytokines. The aim of this present study was to investigate the inflammatory effects and mechanisms of bacteriophage vB_SauM_JS25 in stimulated MAC-T bovine mammary epithelial cells by real-time polymerase chain reaction (PCR) and Western blotting. Experiments show that vB_SauM_JS25 reduces *Staphylococcus aureus*- or lipopolysaccharide (LPS)-induced levels of tumor necrosis factor-α (TNF-α), interleukin (IL)-1β, IL-6, IL-8, IL-10, and regulated on activation, normal T cell expressed and secreted (RANTES) mRNA in MAC-T cells, in a manner expected to be unrelated to its antibacterial action. Moreover, *S. aureus* bacteriophage vB_SauM_JS25 suppressed the LPS-induced phosphorylation of nuclear factor (NF)-κB p65, which may represent an important mechanism mediating these effects. A carefully regulated balance between activation and inhibition by bacteriophages must be kept avoiding inappropriate inflammatory responses. The ability of vB_SauM_JS25 to influence the immune response highlights the potential development and application of bacteriophage-based therapies and may represent a novel anti-inflammatory therapeutic strategy.

## Introduction

Mastitis is defined as inflammation of the mammary gland that principally occurs in response to the invasion of pathogenic microorganisms, such as *Staphylococcus aureus* (Wang et al., 2014). Notably, the invasion of *S. aureus*, and its subsequent effect upon the host’s immune system, is an important cause of mastitis in dairy cows (McDougall et al., 2009). Bacteriophage-based therapy therefore appears to represent a potent and safe alternative tool for the treatment of such bacterial infections.

Data from experimental bacteriophage therapy indicate that successful treatment can correct the increased levels of proinflammatory cytokines associated with bacterial infections both *in vivo* and *in vitro* (Weber-Dabrowska et al., 2000; Zimecki et al., 2009; Hung et al., 2011; Gorski et al., 2012; Secor et al., 2017). Moreover, bacteriophages with both Gram-positive and Gram-negative hosts are able to induce an (innate) immune response *in vitro*. This observed immune response was shown to be endotoxin-independent and predominantly anti-inflammatory (Van Belleghem et al., 2017). However, it is important to consider how bacteriophages can affect the production of cytokines. One mechanism that might be responsible for mediating such effects could be related to the activity of nuclear factor (NF)-κB (Gorski et al., 2006; An et al., 2014).

Previous work has shown that bacteriophages can penetrate MAC-T bovine mammary epithelial cells and are able to clear intracellular *S. aureus* in a time-dependent manner (Zhang et al., 2017). The aim of the present study was to use real-time polymerase chain reaction (PCR) and Western blotting to investigate the inflammatory effects and mechanisms by the virulent of *S. aureus* bacteriophage, vB_SauM_JS25, in MAC-T cells. Our results show that bacteriophages can reduce *S. aureus*- or lipopolysaccharide (LPS)-induced levels of tumor necrosis factor-α (TNF-α), interleukin (IL)-1β, IL-6, IL-10, and regulated on activation, normal T cell expressed and secreted (RANTES) mRNA in MAC-T cells, in a manner that is unrelated to its antibacterial action. Moreover, the current data highlight the fact that the *S. aureus* bacteriophage, vB_SauM_JS25, can suppress the LPS-induced phosphorylation of NF-κB p65.

## Materials and Methods

### Antibodies and Reagents

Anti-phospho-NF-κB p65 (Ser536) (93H1) rabbit monoclonal antibody was purchased from Cell Signaling Technology, Inc. (Beverly, MA, United States). Anti-NF-κB p65 (H-286) and anti-β-actin (C4) were purchased from Santa Cruz Biotechnology (Santa Cruz, CA, United States), while LPS (from *Escherichia coli* O55:B5) was acquired from Sigma (St. Louis, MO, United States).

### Culture of *S. aureus* Strains

*Staphylococcus aureus* strains American Type Culture Collection (ATCC; United States) 6538 and JYG2 were routinely cultivated at 37°C using tryptic soy broth (TSB, QingDao Hope Bio-technology Co., Ltd., Qingdao, China). *S. aureus* ATCC 6538 was routinely used as the host bacterial strain for phage propagation. *S. aureus* JYG2 was isolated from milk samples acquired from a dairy farm and used to infect the MAC-T cells used in this study (Zhang et al., 2016).

### Bacteriophage Preparation

This study used the bacteriophage vB_SauM_JS25, a broad-spectrum virulent bacteriophage belonging to the family *Myoviridae* (Zhang et al., 2015). Stock bacteriophage was prepared using the double-agar overlay method (Han et al., 2013). Phage lysates were further purified using cesium chloride (CsCl) centrifugation to removal toxins, as described previously (Zhang et al., 2015). Then, bacteriophages underwent ultra-filtration through a cellulose membrane (Millipore, Billerica, MA, United States) with a nominal molecular mass limit of 100 kDa to remove the CsCl. Previous work has reported that CsCl gradient ultra-centrifuged bacteriophages are free from residual DNA, RNA, and bacterial proteins released during the lysis of bacterial cells (Reddy et al., 1988).

### Cell Culture and Stimulation

MAC-T bovine mammary epithelial cells were cultured in Dulbecco’s modified Eagle’s medium (DMEM) supplemented with 10% fetal calf serum (Sigma-Aldrich, MO, United States), and were maintained at 37°C with 5% CO_2_. MAC-T cells were then cultured overnight in six-well plates and then infected with *S. aureus* JYG2 (10^6^ bacteria/well) or LPS (1 μg/ml). One or five hours later, cells were treated with purified bacteriophage vB_SauM_JS25 (10^8^ PFU/ml). At the indicated time points, real-time quantitative PCR (Q-PCR) was used to investigate the effects of the bacteriophage on *S. aureus*- or LPS-induced inflammation in MAC-T cells.

### Real-Time Q-PCR

Total RNA was extracted from cellular samples using an RNA kit (OMEGA, Shanghai, China) in accordance with the manufacturer’s instructions. Then, 500 ng of total RNA was used in a 10-μl PrimeScript^TM^ RT Master Mix (TaKaRa, Tokyo, Japan) as described in the manufacturer’s instructions. Next, the cDNA was used as a template for real-time Q-PCR, which was performed on a LightCycler 480 (Roche Diagnostic, IN, United States). The primers used for bovine GAPDH, TNF-α, IL-1β, IL-8, IL-6, IL-10, and RANTES have been described previously (**Table 1**) (Gilbert et al., 2013; Yang et al., 2013; Zbinden et al., 2014). For each sample, data were normalized to the expression level of GAPDH (Zbinden et al., 2014).

**Table 1:** Primers used in this study for real-time quantitative polymerase chain reaction.

Gene	Primer	Sequence 5′–3′	Product size (bp)
GAPDH	Sense	TCAACGGGAAGCTCACTGG	237
	Anti-sense	CCCCAGCATCGAAGGTAGA	
TNF-α	Sense	CCACGTTGTAGCCGACATC	155
	Anti-sense	CCCTGAAGAGGACCTGTGAG	
IL-1β	Sense	AGTGCCTACGCACATGTCTTC	114
	Anti-sense	TGCGTCACACAGAAACTCGTC	
IL-8	Sense	ATGACTTCCAAGCTGGCTGTTG	149
	Anti-sense	TTGATAAATTTGGGGTGGAAAG	
IL-6	Sense	TGCTGGTCTTCTGGAGTATC	153
	Anti-sense	GTGGCTGGAGTGGTTATTAG	
IL-10	Sense	GTGATGCCACAGGCTGAGAA	131
	Anti-sense	TGCTCTTGTTTTCGCAGGGCAG	
RANTES	Sense	GCCAACCCAGAGAAGAAGTG	119
	Anti-sense	CTGCTTAGGACAAGAGCGAGA	


*GAPDH, glyceraldehyde-3-phosphate dehydrogenase; TNF-α, tumor necrosis factor-α; IL-1β, interleukin-1β; IL-8, interleukin-8; IL-6, interleukin-6; IL-10, interleukin-10; RANTES, regulated on activation, normal T cell expressed and secreted.*

### Western Blotting

MAC-T cells were pretreated with bacteriophage vB_SauM_JS25 (10^8^ PFU/well) for 3 h and further incubated with LPS (1 μg/ml) for a further 2 or 5 h. Proteins were then extracted using M-PER mammalian protein extraction reagent (Thermo Fisher Scientific, Waltham, MA, United States) containing protease inhibitors. Whole cell lysates were centrifuged at 14,000 rpm and 4°C for 10 min, and the supernatant was separated by SDS-polyacrylamide gel electrophoresis under reducing conditions. Gels were then transferred onto nitrocellulose membranes that were then blocked with 5% BSA in TBST for 2 h at room temperature. Membranes were then incubated overnight at 4°C with a 1:1000 dilution of each primary antibody in TBST. Each membrane was then washed with TBST and incubated with HRP-conjugated secondary antibody (Abmart). Positive bands were finally visualized with an ECL detection system (Applygen Technologies Inc., Beijing, China) and band intensity quantified using ImageJ software (NIH).

### Statistical Analysis

Data were analyzed for significance by one-way or two-way analysis of variance (ANOVA) using GraphPad PRISM software (version 5.02 for Windows; GraphPad Software, Inc.). A *p*-value <0.05 was considered to indicate a statistically significant difference.

## Results

### Bacteriophage vB_SauM_JS25 Suppressed *S. aureus*-Induced Inflammation in MAC-T Cells

**FIGURE 1:**
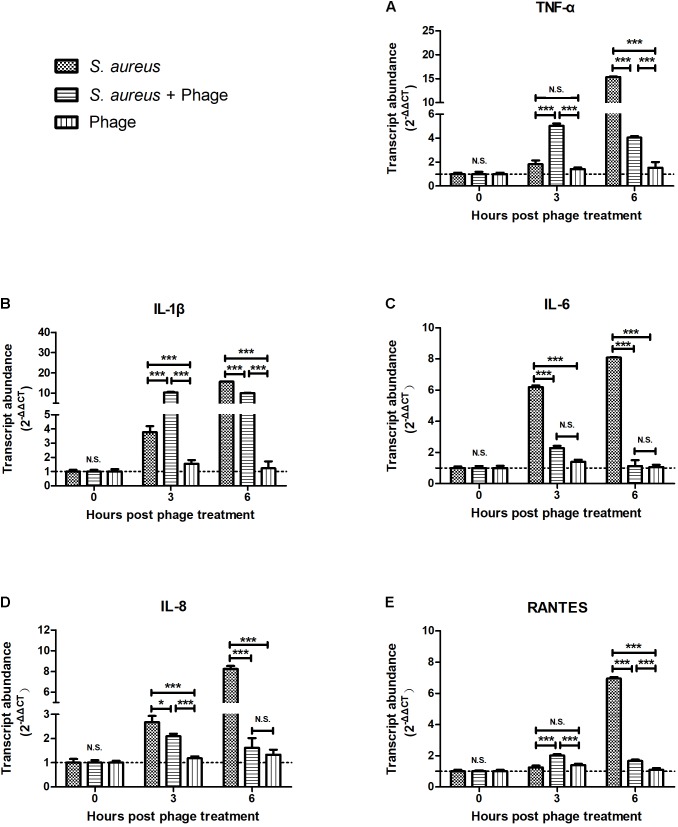
Bacteriophages inhibited the *Staphylococcus aureus*-induced production of inflammatory cytokines and chemokine in MAC-T bovine mammary epithelial cells. MAC-T cells were infected with *S. aureus* JYG2 (10^6^ bacteria/well) and then treated with purified bacteriophage vB_SauM_JS25 (10^8^ PFU/ml). At the indicated time points, the expression of TNF-α **(A)**, IL-1β **(B)**, IL-6 **(C)**, IL-8 **(D)**, and RANTES **(E)** mRNA were determined in MAC-T cells using real-time quantitative polymerase chain reaction. Untreated MAC-T cells were used as a negative control. Data are shown as mean ± standard error of the mean (N.S., not significant, ^∗^*p* < 0.05, ^∗∗^*p* < 0.01, ^∗∗∗^*p* < 0.001 compared to negative control).

To examine whether the bacteriophage affected inflammation, MAC-T cells were first infected with *S. aureus* JYG2. One hour later, cells were washed and treated with highly purified bacteriophage vB_SauM_JS25. As shown in **Figure 1**, the levels of TNF-α, IL-1β, and RANTES in the *S. aureus*-bacteriophage group gradually increased at 3 h post-treatment (h.p.t.) (*p* < 0.001) and then rapidly decreased at 6 h.p.t. (*p* < 0.001) compared to those in the *S. aureus* group. The levels of IL-6 and IL-8 in the *S. aureus*-bacteriophage group were significantly lower than those in the *S. aureus* group (*p* < 0.001). Furthermore, the bacteriophage alone group induced the expression of low levels of cytokines and chemokine.

### Bacteriophage vB_SauM_JS25 Suppressed LPS-Induced Inflammation in MAC-T Cells

**FIGURE 2:**
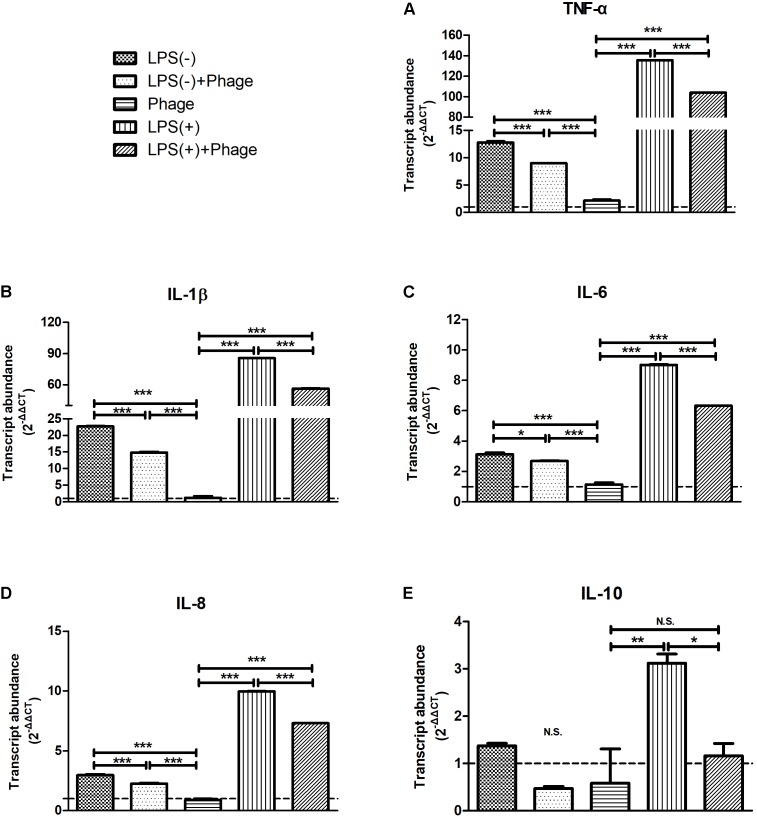
*Staphylococcus aureus* bacteriophage inhibited the lipopolysaccharide (LPS)-induced production of inflammatory cytokines in MAC-T bovine mammary epithelial cells. MAC-T cells were stimulated with LPS (1 μg/ml) for 5 h, and then treated with purified bacteriophage vB_SauM_JS25 (10^8^ PFU/ml) in the absence (–) or presence (+) of LPS for another 3 h. The expression of TNF-α **(A)**, IL-1β **(B)**, IL-6 **(C)**, IL-8 **(D)**, IL-10 **(E)** mRNA in MAC-T cells were determined by real-time quantitative polymerase chain reaction. LPS(–), LPS stimulation for 5 h; LPS(–)+Phage, LPS stimulation for 5 h, bacteriophage treatment for a further 3 h; Phage, bacteriophage treatment for 3 h; LPS(+), LPS stimulation for 5 h, LPS stimulation for another 3 h; LPS(+)+Phage, LPS stimulation for 5 h, LPS plus bacteriophage treatment for another 3 h. Untreated MAC-T cells were used as a negative control. Data are shown as mean ± standard error of the mean (N.S., not significant, ^∗^*p* < 0.05, ^∗∗^*p* < 0.01, ^∗∗∗^*p* < 0.001 compared to negative control).

To further investigate the effects of bacteriophage on inflammation, LPS was used instead of *S. aureus* to exclude the bactericidal action of bacteriophages that might relieve inflammation. MAC-T cells were treated with LPS. Then, 5 h later, these cells were stimulated with bacteriophages in the absence (-) or presence (+) of LPS for another 3 h. We found that bacteriophage vB_SauM_JS25 significantly inhibited the LPS-induced production of cytokines, including TNF-α, IL-1β, IL-6, IL-8 (*p* < 0.001), and IL-10 (*p* < 0.05). Similar results were obtained even in the absence (-) of LPS 5 h post-stimulation (**Figure 2**).

**FIGURE 3:**
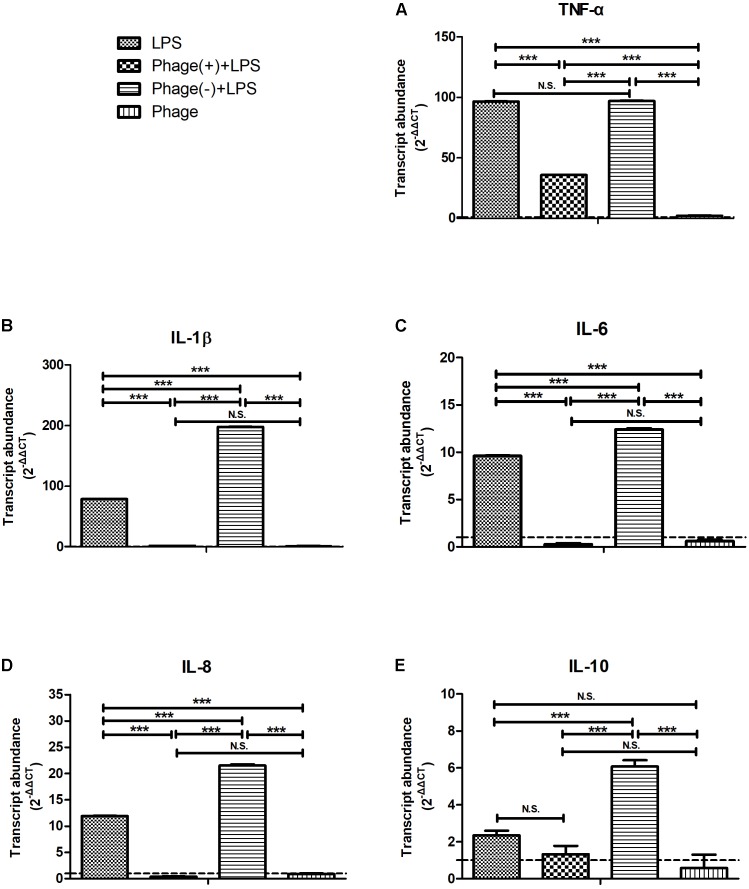
Pre-treatment with bacteriophage did not inhibit the lipopolysaccharide (LPS)-induced production of inflammatory cytokines in MAC-T bovine mammary epithelial cells. MAC-T cells were pre-treated (10^8^ PFU/ml) with bacteriophage for 3 h and then stimulated with LPS (1 μg/ml) in the absence (–) or presence (+) of bacteriophage for another 5 h. The expression of TNF-α **(A)**, IL-1β **(B)**, IL-6 **(C)**, IL-8 **(D)**, and IL-10 **(E)** mRNA in MAC-T cells was then determined by real-time quantitative polymerase chain reaction. LPS, LPS stimulation for 5 h; Phage(+)+LPS, bacteriophage treatment for 3 h, bacteriophage plus LPS treatment for another 5 h; Phage(–)+LPS, bacteriophage treatment for 3 h, LPS stimulation for 5 h; Phage, bacteriophage treatment for 3 h. Untreated MAC-T cells were used as a negative control. Data are shown as mean ± standard error of the mean (N.S., not significant, ^∗^*p* < 0.05, ^∗∗^*p* < 0.01, ^∗∗∗^*p* < 0.001 compared to negative control).

Next, we investigated whether pre-treatment with bacteriophage could inhibit LPS-induced inflammation. MAC-T cells were pre-treated with bacteriophage for 3 h, and then stimulated with LPS in the absence (-) or presence (+) of bacteriophage for another 5 h. As shown in **Figure 3**, consistent treatment with bacteriophage significantly inhibited the LPS-induced production of cytokines (*p* < 0.001). However, the removal of pre-treated bacteriophage significantly enhanced the LPS-induced production of cytokines (*p* < 0.001, **Figure 3**).

### Inhibition of LPS-Initiated NF-κB Signaling in Response to Bacteriophage vB_SauM_JS25

**FIGURE 4:**
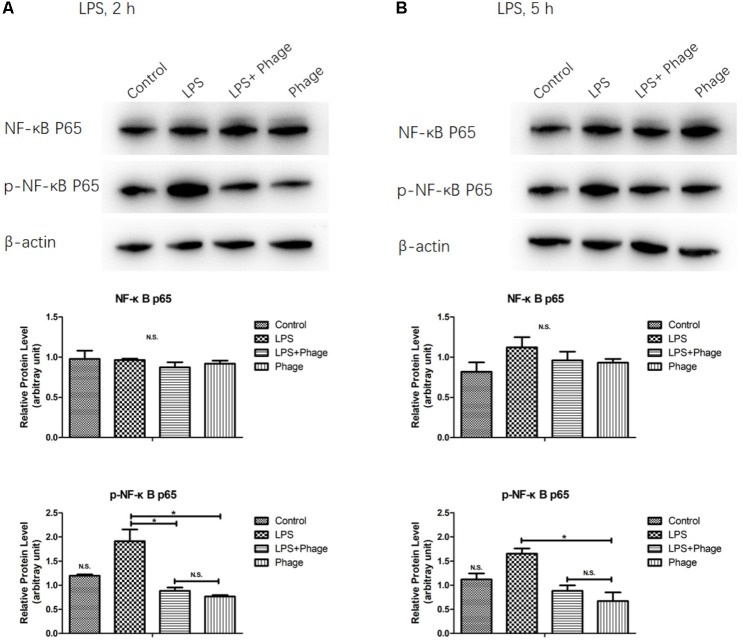
Western blotting analysis showing the inhibition of lipopolysaccharide (LPS)-induced NF-κB activation by *Staphylococcus aureus* bacteriophage. MAC-T cells were pre-treated with bacteriophage vB_SauM_JS25 (10^8^ PFU/well) for 3 h and then further incubated with LPS (1 μg/ml) in the presence of bacteriophage for a further 2 **(A)** or 5 h **(B)**. After immunoblotting, the levels of p65, and the phosphorylation levels of p65, were identified using specific antibodies. Untreated MAC-T cells were used as a negative control. β-Actin was used to ensure equal loading (N.S., not significant, ^∗^*p* < 0.05 compared to negative control).

To investigate the potential involvement of the NF-κB pathway in the bacteriophage-mediated regulation of LPS-induced inflammation, the NF-κB p65 subunit, and its level of phosphorylation, were investigated by Western blotting. As shown in **Figure 4**, LPS increased the levels of NF-κB p65 phosphorylation, but these levels of phosphorylation were significantly suppressed 2 h after LPS stimulation when pre-treated with bacteriophage vB_SauM_JS25 (*p* < 0.05, **Figure 4**). These results imply that the bacteriophage-mediated regulation of LPS-induced inflammation is associated with NF-κB signaling.

## Discussion

Bacteriophage therapy can normalize the levels of inflammatory cytokines associated with bacterial infections both *in vivo* and *in vitro* (Weber-Dabrowska et al., 2000; Zimecki et al., 2009; Hung et al., 2011; Secor et al., 2017). Different bacteriophages, with both Gram-positive and Gram-negative hosts, have been previously shown to induce an immune response, which was endotoxin-independent and predominantly anti-inflammatory. The addition of endotoxins to highly purified bacteriophages did not cause an immune response comparable to the one induced by the (endotoxin containing) bacteriophage lysate (Van Belleghem et al., 2017). It is important to consider whether bacteriophage co-existing with bacteria or toxins might dominantly elicit an antiviral innate immune response or alter the inflammatory response induced by bacteria or toxins. In the present study, we investigated whether the *S. aureus* bacteriophage could affect LPS-induced inflammation in MAC-T bovine mammary epithelial cells, in a manner that was unrelated to its antibacterial action (**Figures 2–4**). A previous study indicated that bacteriophages are able to reduce lung injury in mice infected with *Pseudomonas aeruginosa*, but not due to lower bacterial loads (Secor et al., 2017). Therefore, it is possible that there may be other mechanisms by which bacteriophages directly interact with eukaryotic systems and not just simply by lysing their bacterial hosts.

Several bacterial antigens, known as pathogen-associated molecular patterns (PAMPs), are recognized by pattern recognition receptors on innate immune cells and produce several inflammatory cytokines, such as TNF-α and IL-6 (Sato et al., 2000). In particular, families of Toll-like receptors (TLRs) have been shown to be involved in the recognition of bacterial components (Elson et al., 2007). These recognize and bind a wide variety of bacterial PAMPs, including LPS (typically recognized by TLR4, although some LPS species can be recognized by TLR2), lipopeptides (TLR1, TLR2, and TLR6), lipoarabinomannan and lipoteichoic acid (TLR2 and other TLRs) (Netea et al., 2004). TLR2 and TLR4 are known to play a major role in recognition of Gram-positive and Gram-negative bacteria, respectively (Takeuchi et al., 1999). Research has also shown that TLR2 is a functional receptor for both Gram-positive and Gram-negative bacteria and can induce the activation of IL-8 (Dziarski and Gupta, 2000). The myeloid differentiation factor 88 (MyD88)/NF-κB signal transduction pathway has been shown to be involved in both TLR2- and TLR4-mediated production of inflammatory cytokines (Yamamoto et al., 2002). Therefore, the responses to *S. aureus* and LPS are quite different. In the present study, different incubation times were used for *S. aureus* and LPS prior to bacteriophage treatment (1 and 5 h, respectively). However, it was found that bacteriophage vB_SauM_JS25 suppressed both *S. aureus*- and LPS-induced inflammation in MAC-T cells (**Figures 1–3**). These findings indicate that bacteriophages may affect the common signaling pathway shared by both Gram-positive and Gram-negative bacteria.

Previous studies reported that bacteriophages can diminish cellular infiltration of allogeneic skin allograft in mice, extend its survival and inhibit human T cell activation *in vitro*. Furthermore, T4 phage can abolish the ability of the pathogenic virus to induce NF-κB activity (Gorski et al., 2006). In order to prove the relationship between the effects induced by bacteriophages and NF-κB, we determined the expression levels and the phosphorylation of the NF-κB p65 subunit were determined by Western blotting. This part of our work demonstrated that pre-treatment with bacteriophage vB_SauM_JS25 significantly suppressed the phosphorylation levels of NF-κB p65 at 2 h post-LPS-stimulation (*p* < 0.05, **Figure 4**). However, once the pre-treatment bacteriophage was removed, the LPS-induced production of cytokines was significantly enhanced (*p* < 0.001, **Figure 3**). As reported previously, the lack of dissemination, and the reduced levels of inflammation caused by the production of prophage-created conditions, could promote persistent infection by *P. aeruginosa* (Secor et al., 2017). Moreover, there may be other mechanisms that bacteriophages use to interact directly with eukaryotic systems and thus modulate the immune system. In these scenarios, bacteriophages appear to act an immunomodulator in order to balance inflammation cytokines.

In this study, the levels of TNF-α, IL-1β, and RANTES mRNA in the *S. aureus*-bacteriophage group gradually increased at 3 h.p.t. and then rapidly decreased at 6 h.p.t. compared to those in the *S. aureus* group. This may be the reason for the greater level of toxin exposure when the bacteria were lysed, the same way as antibiotic does (Holzheimer, 2001). Bacteriophages can then bind to toxins and counteract toxin-induced inflammation, such as T4 gp12-LPS (Miernikiewicz et al., 2016). Another possibility may be an increase in bacteriophage concentration following the replication in *S. aureus*. Moreover, there are other mechanisms by which bacteriophages directly interact with eukaryotic systems. The transcription factor NF-κB has been recognized as a frequent target for immunosuppressive and anti-inflammatory molecules (Baeuerle and Baichwal, 1997). Our current experiments demonstrated that pre-treatment with bacteriophage significantly suppressed the phosphorylation levels of NF-κB p65 at 2 h post-LPS-stimulation (*p* < 0.05, **Figure 4**). In accordance with the results shown in **Figure 3**, bacteriophage treatment significantly inhibited the LPS-induced production of cytokines (*p* < 0.001) in the Phage(+)+LPS group. Moreover, when cytokine levels were reduced by bacteriophage treatment in the Phage(+)+LPS group at 5 h post-LPS-stimulation (**Figure 3**), there was no significant difference in the phosphorylation levels of NF-κB p65 when compared between the LPS and LPS+Phage group at the same time point (**Figure 4**). Previous studies have indicated that bacteriophage therapy can correct abnormal levels of inflammatory cytokines (Weber-Dabrowska et al., 2000; Zimecki et al., 2009; Hung et al., 2011). It appears that bacteriophage will not continuously suppress the phosphorylation levels of NF-κB p65 in order to reduce inflammation. Therefore, in our current-case, bacteriophage vB_SauM_JS25 could not promote a persistent infection simply by promoting the survival of bacteria. The ability of vB_SauM_JS25 to influence the immune response highlights the potential development and application of bacteriophage-based therapies and may represent a novel anti-inflammatory therapeutic strategy for mastitis.

Collectively, the results described herein suggest that *S. aureus* bacteriophages can reduce inflammation in MAC-T bovine mammary epithelial cells in a manner unrelated to its antibacterial action. Furthermore, *S. aureus* bacteriophages can suppress the LPS-induced phosphorylation of NF-κB p65, which may represent one of the mechanisms that mediates such effects. In an earlier study, it was found that the bacteriophage vB_SauM_JS25 was able to cross MAC-T cell membranes and reach the nucleus (Zhang et al., 2017). Our current observations further implicate phage vB_SauM_JS25 as a potential means of reducing infection. The mechanism by which bacteriophages affect the immune response in MAC-T cells will now need to be further elucidated.

## Author Contributions

LZ and RW designed the experiments. LZ, XH, LS, TH, RCW, and MP performed the experiments. LZ, XH, LS, and TH analyzed the data. TH, RCW, and MP contributed reagents, materials, and analysis tools. LZ wrote the paper.

## Conflict of Interest Statement

The authors declare that the research was conducted in the absence of any commercial or financial relationships that could be construed as a potential conflict of interest.
