# Corrigendum: *Staphylococcus aureus* Bacteriophage Suppresses LPS-Induced Inflammation in MAC-T Bovine Mammary Epithelial Cells

**DOI:** 10.3389/fmicb.2018.02511

**Published:** 2018-10-19

**Authors:** Lili Zhang, Xiang Hou, Lichang Sun, Tao He, Ruicheng Wei, Maoda Pang, Ran Wang

**Affiliations:** Key Laboratory of Food Quality and Safety of Jiangsu Province-State Key Laboratory Breeding Base, Institute of Food Safety and Nutrition, Jiangsu Academy of Agricultural Sciences, Nanjing, China

**Keywords:** *Staphylococcus aureus*, bacteriophage, inflammation, LPS, NF-κB, MAC-T cells

In the original article, there was an error. We stated in the article that Previous studies reported that bacteriophages could activate NF-κB signaling and enhance immune effects *in vitro* (Gorski et al., 2006). In fact, the authors of the aforementioned article reported the exact opposite; that is, they reported a phage-mediated down-regulation of NF-κB activation.

A correction has been made to Discussion, Paragraph 3:

Previous studies reported that bacteriophages can diminish cellular infiltration of allogeneic skin allograft in mice, extend its survival and inhibit human T cell activation *in vitro*. Furthermore, T4 phage can abolish the ability of the pathogenic virus to induce NF-κB activity (Gorski et al., 2006). In order to prove the relationship between the effects induced by bacteriophages and NF-κB, we determined the expression levels and the phosphorylation of the NF-κB p65 subunit were determined by Western blotting. This part of our work demonstrated that pre-treatment with bacteriophage vB_SauM_JS25 significantly suppressed the phosphorylation levels of NF-κB p65 at 2 h post-LPS-stimulation (*p* < 0.05, **Figure 4**). However, once the pre-treatment bacteriophage was removed, the LPS-induced production of cytokines was significantly enhanced (*p* < 0.001, **Figure 3**). As reported previously, the lack of dissemination, and the reduced levels of inflammation caused by the production of prophage-created conditions, could promote persistent infection by *P. aeruginosa* (Secor et al., 2017). Moreover, there may be other mechanisms that bacteriophages use to interact directly with eukaryotic systems and thus modulate the immune system. In these scenarios, bacteriophages appear to act an immunomodulator in order to balance inflammation cytokines.

The authors apologize for this error and state that this does not change the scientific conclusions of the article in any way.

The original article has been updated.

## Conflict of interest statement

The authors declare that the research was conducted in the absence of any commercial or financial relationships that could be construed as a potential conflict of interest.
